# Sympathetic ophthalmia in HIV infection. A clinicopathological case report

**DOI:** 10.1007/s12348-012-0065-y

**Published:** 2012-03-13

**Authors:** Miguel A. de la Fuente, Nicolas Alejandre, Patricia Ferrer, Gillermo Fernandez, Jose L. Sarasa, Olga Sanchez

**Affiliations:** 1 Department of Ophthalmology, Fundacion Jimenez Diaz University Hospital, Avda Reyes Catolicos 2, 28040 Madrid, Spain; 2 Department of Pathology, Fundacion Jimenez Diaz University Hospital, Avda Reyes Catolicos 2, 28040 Madrid, Spain; 3 Department of Rheumatology, Fundacion Jimenez Diaz University Hospital, Avda Reyes Catolicos 2, 28040 Madrid, Spain; 4 Avda del Valle 13, 28003 Madrid, Spain

**Keywords:** Sympathetic ophthalmia, Panuveitis, AIDS, HIV

## Abstract

**Background:**

The purpose of this study is to report a case of sympathetic ophthalmia (SO) in an HIV-infected patient on treatment with highly active antiretroviral therapy (HAART) 9 years after a penetrating eye injury.

**Methods:**

The study utilized clinical course and histopathological findings.

**Results:**

Histopathology of the enucleated right eye showed a predominantly lymphocytic inflammatory infiltration with some plasma cells and epithelioid granulomata in the choroid, suggesting the diagnosis of SO.

**Conclusions:**

SO seems to be driven by T lymphocytes, specifically by the CD4 subset of T cells. HIV-infected individuals suffer a decline in CD4 T cell numbers, leading to an acquired immunodeficiency that could halt the development of the inflammatory reaction responsible for SO. The restoration of the CD4 counts by HAART therapy makes HIV-infected individuals as susceptible to SO as non-infected ones. To the best of our knowledge, there are no cases of SO in HIV-infected patients reported in the literature.

## Introduction

Sympathetic ophthalmia (SO) is a rare, bilateral, non-necrotizing, granulomatous panuveitis that occurs after ocular surgery or trauma to one eye threatening sight in the uninjured fellow eye. The existence of the disease has been known since Hippocrates; however, it was the Scottish ophthalmologist Sir William MacKenzie who provided the first clinical description and named the disease as *sympathetic ophthalmitis.* Later on, Ernst Fuchs described the disease and its histopathology [1].

Kilmartin et al. reported a SO incidence of 0.03 in 100,000 persons. Traditionally, accidental penetrating eye injury was considered the main risk of SO. Nowadays, ocular surgery, particularly vitrectomy for retinal detachment, is also recognized as a risk factor for SO [2]. There is equal incidence of SO in men and women. There is no racial predisposition and SO has been reported in all age groups [3]. Presentation has been reported from 1 week to 66 years after initial injury [4] with a median time interval of 12 months [2].

The etiology of SO remains elusive. A recent hypothesis proposes that SO results from an autoimmune, inflammatory response against ocular antigens located in the uveal tissue, retina, or choroidal melanocytes exposed to the lymphatic system of conjunctiva and orbit [5]. Immunologic studies of SO specimens have shown CD4 helper and inducer T cells during the early phase of inflammation, with infiltration by CD8 suppressor and cytotoxic T cells in the later stages [6].

We present herein a histopathologically supported case of SO after a penetrating ocular injury in one eye of a patient with HIV infection on treatment with antiretroviral medication. A Medline search for cases of SO and HIV/AIDS retrieved no results.

## Case report

A 39-year-old Caucasian man presented to the department of ophthalmology with severe loss of vision in the left eye (LE) over the last month. His past ocular history was relevant for an accidental penetrating injury to the right eye (RE) 9 years before. He was an intravenous drug addict diagnosed with HIV and chronic hepatitis C 20 years previously. He was on treatment with the highly active antiretroviral therapy (HAART) combination emtricitabine, tenofovir, and nevirapine. Unfortunately, previous CD4 counts and medical records could not be obtained.

On examination, visual acuity (VA) was no light perception in the RE and hand movements in the LE. The RE was in phthisis bulbi. LE showed moderate ciliary injection and mutton fat keratic precipitates. There were +3 cells and +3 flare in the anterior chamber with multiple posterior synechiae. There was severe vitritis with no fundus view. The patient was admitted for further work-up and treatment. Blood samples analyses taken upon admission showed a CD4+ T lymphocyte count of 549 with undetectable HIV viral load. Our working differential diagnoses were SO, acute retinal necrosis, toxoplasma panuveitis, syphilitic or tuberculous panveitis, sarcoidosis, endogenous endophthalmitis and less likely, immune reconstitution inflammatory syndrome (IRIS). Chest x-ray and head CT scan were reported as normal. Treatment was initiated with topical dexamethasone 0.1 % and atropine 1 %. Systemic treatment was started with oral valacyclovir, intravenous (IV) 1 g methylprednisolone daily for 3 days, followed by oral prednisone 1 mg/kg daily. Syphilis and toxoplasma serology were negative. QuantiFERON®-TB Gold (Cellestis Ltd. Carnegie, Victoria, Australia) was negative. Polymerase chain reaction (PCR) of aqueous and vitreous taps for herpes simplex virus 1 and 2 and varicella zoster virus was negative. PCR for CMV was not done. In view of the blood analyses and PCR results, valacyclovir was discontinued.

**Fig. 1:**
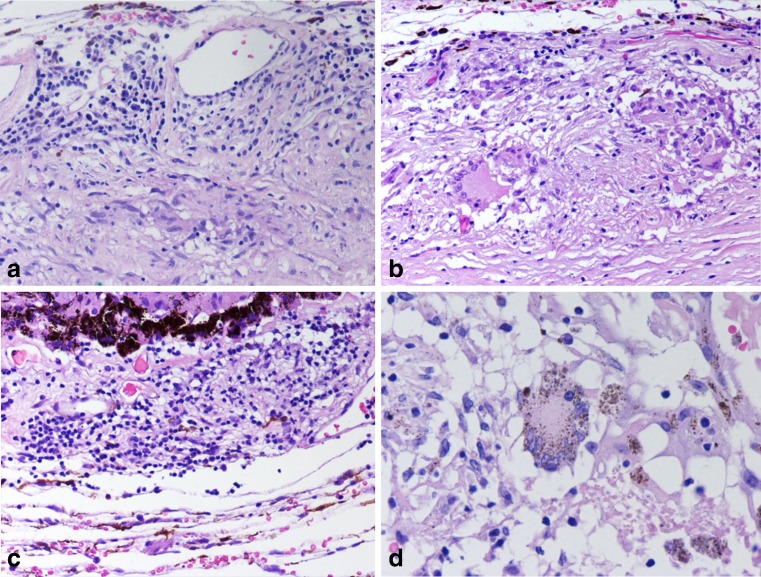
Sympathetic ophthalmia. **a** Photomicrograph of a choroidal section showing extensive inflammatory infiltration predominantly by lymphocytes with some plasma cells and choroidal granuloma composed mainly of epithelioid cells (Dalen-Fuchs nodules; Hematoxylin and eosin ×200). **b** Photomicrograph of a choroidal section showing epithelioid granulomata and multinucleated giant cell (Hematoxylin and eosin ×200). **c** Photomicrograph of a choroidal section showing a dense lymphocytic infiltration (Hematoxylin and eosin ×200). **d** Photomicrograph of a choroidal section of enucleated inciting right eye depicting a multinucleated giant cell with phagocytosed melanin granules (Hematoxylin and eosin ×400)

Enucleation of the phthisical RE was performed. Microscopically, the choroid presented foci of lymphocytes and plasma cells as well as epithelioid granulomata with some multinucleated giant cells (Fig. 1a–c). These multinucleated giant cells contained phagocytosed melanin granules (Fig. 1d). There was fibrosis between the choroid and retina with large cholesterol deposition and groups of epithelioid cells interpreted as Dalen-Fuchs nodules. After 3 months of therapy with oral prednisone, the alkylating agent cyclophosphamide at a dose of 25 mg daily was added as steroid-sparing medication.

**Fig. 2:**
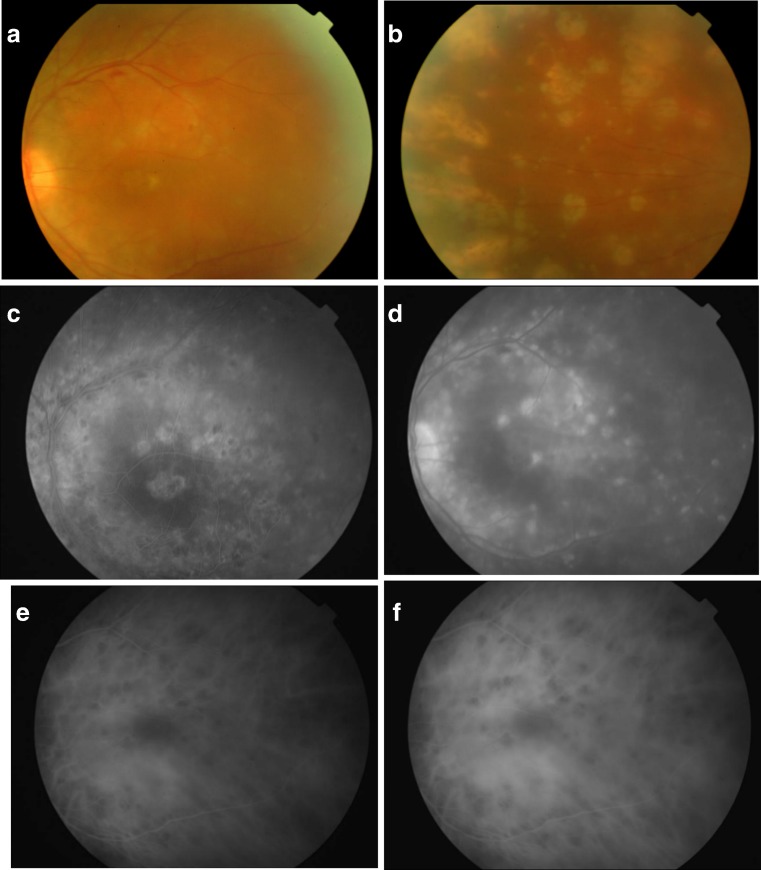
Sympathetic ophthalmia composite of color, fluorescein angiography (FA), and ICG of sympathizing left eye. **a**, **b** Fundus photographs showing widespread retinal pigment epithelium depigmented lesions with a small intraretinal hemorrhage along the superior arcade. **c** Early-phase FA revealing multiple diffuse hypofluorescent choroidal lesions. **d** Late-phase FA showing the choroidal lesions becoming hyperfluorescent. **e**, **f** ICG early and late phases depicting widespread hypofluorescent choroidal lesions (Dalen-Fuchs nodules)

As the inflammation subsided, fundus examination of the LE showed moderate vitritis with widespread retinal pigment epithelium patches of depigmentation and a small intraretinal hemorrhage along the supero-temporal vascular arcade. There were no signs of vasculitis (Fig. 2a, b). Fluorescein angiography (FA) revealed multiple diffuse hypofluorescent choroidal lesions (Dalen-Fuchs nodules) in early frames that became hyperfluorescent in late phases (Fig. 2c, d). Indocyanine green angiography (ICG) depicted multiple hypofluorescent choroidal lesions in early and late frames widespread over the fundus (Fig. 2e, f).

At 15-month follow-up, VA was 20/200 with ongoing recalcitrant intraocular inflammation. Cyclophosphamide was discontinued, and a new 3-day pulse of 1 g methylprednisolone IV was administered followed by oral prednisone 1 mg/kg daily and cyclosporine A at a dose of 7 mg/kg/daily.

## Discussion

We present herein a histopathologically supported case of SO in an HIV-infected patient on HAART therapy 9 years after a penetrating injury to his RE. The definition of SO is that of a rare, bilateral, non-necrotizing, granulomatous panuveitis that occurs after ocular surgery or trauma to one eye (inciting eye) threatening sight in the uninjured fellow eye (sympathizing eye) [1]. Until the study of Kilmartin et al., it was the general belief that accidental perforating eye injury was the main predisposing risk for SO; however, these authors suggested that ocular surgery, mainly vitrectomy for retinal detachment surgery, was the most important risk factor for the disease in their study [2]. Immunological studies have implicated CD4+ T lymphocytes as the main drivers of SO inflammation, although CD8+ T cells and B lymphocytes have also been reported [6]. T cell-driven autoimmune diseases can occur in HIV carriers even in the presence of very low T cell counts. Psoriasis is an example and illustrates how HIV favors immune dysregulation [7]. HAART therapy leads to restoration of T cell functions with general improvement in patient’s health. However, in up to 25 % of patients, a disturbance in the reconstitution of T cells can provoke inflammatory syndromes. Apparently, these paradoxical events result from the abnormal expansion of selective CD4+ T cell clones targeting antigens of previous opportunistic infections [8]. Although SO is not induced, as far as we know, by infectious antigens, its pathogenesis is not much different from that of IRIS syndrome. In this regard, the upregulation of innate responses and the positive selection of auto-reactive melanocyte-specific T cells are characteristic of T cells. These could suggest that HIV exerts a negative regulation of immune surveillance of both infectious and endogenous antigens that is lost with HAART therapy [9]. HIV infection leads to a fall in the numbers of CD4+ T cells with the subsequent acquired immunodeficiency. A direct cause–effect relationship between restoration of CD4 count and SO cannot be made on the basis of only one case and lacking previous CD4 counts. We speculate that the deficit of T cells could have impaired the buildup of an inflammatory reaction and could have halted the development of SO. The restoration of the CD4 counts by HAART therapy makes HIV-infected individuals as susceptible to SO as non-infected ones.
